# Caesarean delivery: Bringing more than just a bundle of joy

**DOI:** 10.1080/24740527.2019.1574538

**Published:** 2019-07-30

**Authors:** Jane Quinlan

**Affiliations:** Nuffield Division of Anaesthetics, John Radcliffe Hospital, Oxford University Hospitals Trust, Oxford, UK

**Keywords:** caesarean delivery, caesarean section, C-section, pain, postoperative, chronic pain, pregnancy, estradiol, progesterone, depression, postpartum, breastfeeding, resilience, psychological, sleep deprivation

## Abstract

**Background**: Chronic postsurgical pain (CPSP) is a potential complication of all surgical procedures, including caesarean delivery (CD). Psychological factors such as anxiety and depression with negative coping strategies are known to increase the risk of the development of CPSP.

**Aim**: This short review will appraise some additional features that are unique to CD and assess the interplay between them and persisting pain.

**Methods**: This is a narrative review with a focus on describing relevant features associated with the development of CPSP after CD.

**Results**: Hormone changes, postpartum depression, breastfeeding, and sleep disturbance each may affect the mother’s pain in the short and long term. Together they have the potential to negatively impact the mother–infant bond.

**Conclusions**: In the weeks after delivery, pain, depression, and poor sleep negatively impact the mother’s quality of life and her ability to care for, and bond with, her baby. This represents a critical time for the emotional, social, and behavioral development of the infant. Far-reaching benefits for the whole family may be realized by the early identification and management of persisting postoperative pain and postpartum depression.

## RÉSUMÉ

**Contexte**: La douleur chronique postopératoire est une complication possible de toutes les interventions chirurgicales, y compris l’accouchement par césarienne. Il est bien connu que certains facteurs psychologiques comme l’anxiété et la dépression, assortis de stratégies d’adaptation négatives, augmentent le risque de souffrir de douleur chronique postopératoire.

**But**: Ce bref examen porte sur certaines caractéristiques particulières de l’accouchement par césarienne et évalue l’interaction entre celles-ci et la douleur persistante.

**Méthodes**: Un examen narratif mettant l’accent sur la description des caractéristiques pertinentes associées au développement de la douleur chronique post-chirurgicale après un accouchement par césarienne.

**Résultats**: Les changements hormonaux, la dépression postpartum, l’allaitement et les perturbations du sommeil peuvent tous affecter la douleur de la mère à court terme et à long terme. Ensemble, ils peuvent avoir un effet négatif sur le lien entre la mère et le nourrisson.

**Conclusions**: Dans les semaines suivant l’accouchement, la douleur, la dépression et les problèmes de sommeil peuvent avoir des répercussions négatives sur la qualité de vie de la mère, ainsi que sur sa capacité à s’occuper de son bébé et à créer un lien avec lui. Il s’agit d’un moment de première importance pour le développement émotionnel, social et comportemental de l’enfant. La détection précoce et la prise en charge de la douleur postopératoire persistante ainsi que de la dépression postpartum peuvent avoir des bienfaits de grande portée pour toute la famille.

Caesarean delivery (CD) is one of the most commonly performed operations, accounting for one in every 14 surgical procedures carried out worldwide. Globally, the rates of caesarean delivery have almost doubled from 12% of all births in 2000 to 21% in 2015, with huge regional variation: Latin America and the Caribbean have rates of CD up to ten times higher than those in west and central Africa.^1^

Chronic pain after surgery was first described in 1998,^2^ and the ensuing 20 years have brought greater understanding of its incidence, characteristics, and precipitants. Various contributing factors for chronic postsurgical pain (CPSP) have been postulated, with more attention now paid to the impact of patient-specific, surgery-specific, and pain-specific factors, with a growing recognition that the interplay of multiple factors is likely to be complex.

The incidence of chronic pain after CD has been variously documented from 8% to 18% at 3 months, to 0% to 18% at 1 year postpartum, with those studies investigating multiple time points identifying a reduction in the rate and severity of chronic pain over time.^3–7^ Of note, the study finding the lowest incidence of pain at 1 year used a study design that only followed up those who described pain at the earlier time points of 2 and 6 months; those who were pain free at any time point were not included in further data collection.^7^ This assumes that CPSP is an ongoing, linear process with persistent pain from the time of surgery. In fact, some long-term postsurgical pain develops after a seemingly pain-free period, so this study may underestimate the incidence by missing those with a nonlinear trajectory.^8^ Most women with chronic pain after CD describe the pain as mild and precipitated by movement. Although reassuring that the pain is not severe, its impact on quality of life should not be underestimated: population studies show that the presence of any chronic pain is associated with a decreased return to work, interference with daily activities, and poorer general health.^9^ Thus, though the incidence and severity of CPSP after CD may not be as high as, for example, that after breast cancer treatment, the high volume of surgery potentially contributes to a significant societal burden of pain.

It is well recognized that psychological factors such as anxiety, depression, and catastrophizing can increase the risk of chronic pain development after all types of surgery.^10^ CD presents unique features that may also influence the development of chronic pain and merit further investigation. These additional elements are being explored in more detail in the current Oxford Persisting Post-Operative Pain Study (OxPPOPS) examining the incidence and predictive factors of chronic pain after CD. This is an observational prospective study of 728 women undergoing elective CD in Oxford, UK, investigating the incidence of long-term pain 1 year after elective CD, with the secondary outcome to identify predictive factors of this chronic pain. The study protocol encompasses the standard core risk factor and outcome domains for CPSP studies as recommended by VanDenKerkhof et al.^11^ Specifically, participants completed a preoperative questionnaire collecting information about demographics, psychology, pain, and expectations, with a similar questionnaire completed at 4 and 12 months postdelivery. Anesthetic and surgical data were collected at the time of surgery, and blood was taken for hormone analysis. Pain in the early postoperative period was assessed using face-to-face or telephone interviews at 6 h and at days 2, 7, and 30.

In addition, factors specific to pregnancy and the postpartum period were explored. The following is not intended to be a comprehensive review but instead discusses the possible impact of some of these factors.

## Hormones

Pregnancy is a high-progesterone, high-estrogen state, with levels of each up to ten times those seen during a menstrual cycle.

Studies of the impact of menstrual hormone changes on pain perception have found that high levels of progesterone, in the presence of high estradiol, produce a dissociation of the subjective pain rating and its unpleasantness such that, although pain is recognized, it is perceived as less distressing.^12^ Investigation of this effect using functional magnetic resonance imaging techniques reveals a decreased connectivity between the inferior frontal gyrus (IFG), a region of the prefrontal cortex, and the amygdala.^12^ The IFG has been found to modulate pain unpleasantness and links both to the nucleus accumbens, generating positive reappraisal of emotion, and to the amygdala, generating negative appraisals.^13^ This uncoupling of the IFG to the amygdala in the high-progesterone, high-estradiol state thus reduces the negative emotional bias applied to painful stimuli. As visceral pain involves a higher affective component than somatic pain, this may explain why the intra-abdominal discomfort caused by the expanding uterus in pregnancy is not perceived as unpleasant pain.

The delivery of the baby then precipitates an abrupt fall in progesterone and estrogen to levels equivalent to those in the early menstrual phase.

## Postnatal depression

The global prevalence of postpartum depression (PPD) is estimated at 17.7%, with international variations ranging from 3% in Singapore to 38% in Chile.^14^ Although PPD is assumed to develop within the first few weeks or months following delivery, those with the most severe PPD have often already experienced symptoms during pregnancy.^15^ The rapid decrease in reproductive hormone levels has been suggested as one of the causes of PPD in vulnerable women, and identified risk factors include a history of anxiety or depression, stressful life events, previous abuse, low social support, and low income.^16^ For chronic pain generally, there is a clear interplay between pain and depression, with each likely to exacerbate the other. Following CD, severe acute pain within the first 36 h of delivery has been shown to increase the risk of depression as well as continued pain at 6 weeks.^7^ Similarly, women with perineal pain that persists for 4–6 weeks postdelivery had an increased risk of depression at both 4–6 weeks and 6 months.^17^

## Breastfeeding

Postpartum depression can negatively impact breastfeeding duration, with the median duration of breastfeeding in those with early onset PPD being 26 weeks compared to 39 weeks in women with no depression, as reported in an Australian study.^18^ Conversely there is also some evidence that exclusive breastfeeding in the first 3 months postpartum may reduce symptoms of depression, possibly because the enhanced mother–child interaction directly leads to improved maternal mental health^19^ and the release of oxytocin and prolactin, the hormones responsible for lactation, promote relaxation and attenuate stress responses, thus improving mood.^20^ Interestingly, oxytocin, with its regulatory effects on pain, mood, and stress responses, has been postulated as a novel analgesic for chronic pain.^21^ Breastfeeding brings well-known positive health benefits to the infant, particularly protection against childhood infections, together with claims of higher intelligence, fewer emotional and behavioral difficulties in later life, and protection against obesity and diabetes, although many of these outcomes are confounded by socioeconomic factors and thus may reflect association rather than causation.^22^

Ongoing pain can affect breastfeeding in two ways: women may be reluctant to take analgesia for fear of drug transfer to their baby during breastfeeding leading to poorly managed pain, and abdominal pain from the caesarean scar may compromise positioning, making feeding more difficult.^23^

Occasionally breastfeeding itself can worsen perinatal mood and anxiety disorders. Difficulties with breastfeeding can contribute to a sense of failure, and the frequent nighttime interruptions to feed prevent restorative sleep, which is particularly important in mood stabilization.^24^

## Sleep disturbance

Sleep disturbances after surgery result in more severe pain and a higher analgesic requirement, together with a slower recovery.^25^ Patients who slept poorly in the first few weeks after a total knee replacement had worse functional outcomes at 3 months compared to their more rested counterparts. Sleep and pain have a bidirectional effect: pain interrupts sleep, and fragmented sleep increases pain sensitivity.^26^ Repeated awakenings, in contrast to simple sleep restriction, appear to reduce endogenous pain inhibition and produce spontaneous pain.^27^

A study in Norway^28^ investigated the impact of sleep interruptions from a newborn baby on the development of depression in postpartum women and found that poor sleep was associated with a higher risk of depression. Poor sleep is also associated with decreased resilience,^29^ which has three aspects to it: the ability to (1) face and cope with challenges, (2) adapt to the changes created by these challenges, and (3) recover and even grow from these setbacks.^30^ Resilience is now a concept used in pain medicine to explain effective coping with chronic pain, reflecting positive behaviors and attitudes.^31^

## Becoming a mother

A new baby presents many challenges and requires resilience at a time when this may be lacking. The “Being a Mother” questionnaire designed by Matthey^32^ addresses the many conflicting aspects of new motherhood: the joy, excitement, anxiety, and exhaustion. The questionnaire uses Likert scales to cover social isolation, regret, sense of confidence, relationship with the child, satisfaction with support, coping, and guilt, and is scored from 0 to 39, with the higher scores representing least satisfaction with motherhood. Matthey divides the questions into three sections: the Child Experience, which focuses on the mother’s experience with her baby; the Adult’s Experience, asking whether she feels lonely or bored or misses her previous life; and Emotional Closeness, addressing her bonding with the baby. Different factors emerge as important depending on the child’s age. Mothers of babies (0–3 months old) reported feeling nervous around their baby more commonly than mothers of 3- to 12-month-olds, while those with older children aged 1 year and older were more likely to feel unsupported or felt unable to ask for help compared to mothers of younger children. This provides a broad view of how a woman is coping and viewing her experience as a parent, so can be used in conjunction with the more specific Edinburgh Postnatal Depression Scale.^33^ Interrupted sleep and pain will reduce resilience, which may affect the Child Experience domains of “feeling confident about looking after my baby,” “finding it hard to cope when my baby cries,” and “feeling annoyed or irritated with my baby.”

## Conclusion

When investigating CPSP after most procedures, the focus is often on the long-term view: the continuation of pain for many months or years after surgery and the impact on the patient’s quality of life. However, for persisting pain after CD, the first few weeks and months represent an additional crucial period. The intricate and intertwined relationship between pain, depression, and sleep will have consequences for both the mother’s quality of life and her ability to care for and bond with her baby, at a time that is critical for the emotional, social, and behavioral development of the infant.

We can reduce the risk of CPSP by ensuring optimal pain management for all patients in the first hours and days after surgery. We should look to identify women preoperatively who have psychological risk factors for the development of CPSP and provide them with support to shift from negative to positive coping strategies.^34^ Postoperatively, the early identification of persisting pain and postpartum depression, with interventions to ameliorate them, thus has the potential to provide far-reaching benefits for the whole family.
